# Research progress on the diagnostic techniques and vaccine development of bovine herpesvirus types 1

**DOI:** 10.3389/fvets.2025.1703336

**Published:** 2025-10-29

**Authors:** Jianming Zhang, Jian Cao, Liang Cao, Chao Zhang, Liyuan Gong, Xiaohong Kang, Dehong Li, Yang Zhang

**Affiliations:** College of Bioengineering, Jiuquan Vocational and Technical University, Jiuquan, China

**Keywords:** bovine herpesvirus type 1, cattle, diagnostic approaches, vaccine development, prevention and management

## Abstract

Bovine herpesvirus type 1 (BoHV-1) constitutes a major etiological agent associated with the onset of bovine respiratory disease in cattle. This virus is widely prevalent across global cattle populations, leading to significant economic losses. The advancement of early diagnostic methodologies and the formulation of effective vaccines are crucial for the prevention, control, and potential eradication of BoHV-1. This review offers a comprehensive overview of current diagnostic methodologies for BoHV-1, encompassing serological diagnostic approaches, molecular detection methods, and emerging novel techniques. Furthermore, recent development in vaccine research targeting BoHV-1 infection are discussed. Ultimately, this review aims to provide critical insights into the management of BoHV-1 infection, and guide future research efforts in the enhancement of diagnostic tools and vaccine strategies.

## 1 Introduction

Infectious bovine rhinotracheitis (IBR), attributed to bovine herpesvirus type 1 (BoHV-1), represents a significant infectious disease that results in substantial economic losses within the bovine industry (1). The disease caused by BoHV-1 infection has been listed a notifiable animal disease by the World Organization for Animal Health (WOAH). In addition to IBR, the clinical symptoms of this disease are characterized by reproductive disorders and the reduction in milk production in cows and central neural disease in calves (2). Moreover, BoHV-1 infection can also cause immunosuppression in cattle, thus the co-infection with BoHV-1 and other pathogens have been frequently monitored in clinical samples (3).

In the early 1940s, the initial documented case of BoHV-1 infection, referred to as bullous vaginitis, was identified in cattle within the United States. Following this, specific antibodies targeting BoHV-1 were detected in cattle in 1941 and the etiological agent (BoHV-1) was successfully isolated from clinical specimens in 1955 (4). Currently, BoHV-1 exhibits a high prevalence across various regions, including Europe (notably Ireland (5), Serbia (6), Iraq (7), and Irish (8)), Asia (specially Turkey (9) and Iran (10)), as well as the United States (11). In addition to cattle, this pathogen is capable of infecting a variety of mammals, including goats (12, 13) and deer (14, 15), thereby drawing significant attention to its potential threat to other animal industry.

Considering the persistent prevalence of BoHV-1 in cattle herds, the advancement of timely and accurate diagnostic approaches, as well as effective vaccines, is imperative for effective disease management and eradication. This review comprehensively synthesizes current advancements in diagnostic techniques and vaccine development targeting BoHV-1 infection, with the objective of establishing a foundational framework to support the prevention, control, and prospective elimination of this disease.

## 2 Genomic characteristics

BoHV-1 is an enveloped, double-stranded linear DNA virus that belongs to the members of *Alpha-herpesvirinae* subfamily (16). The DNA genome of BoHV-1 contains many open reading frames, which encode various structural proteins and regulatory proteins (17). The genome of BoHV-1 encodes multiple glycoproteins, including gL, gM, gB, gC, gD, and gE, etc., Among which, gB, gC, and gD play critical roles in viral entry into host cells and elicit the production of virus-neutralizing antibodies (18–20). The gE, gG, and the thymidine kinase (TK) enzyme are closely associated with virus virulence, deletion of these genes significantly attenuates viral pathogenicity without compromising viral replication or immunogenicity. Consequently, these genes are frequently targeted for the development of gene-deleted vaccines and differential diagnostic assays (21, 22).

According to the genetic characteristics of BoHV-1 strains, which are classified into three subtypes: BoHV-1.1, BoHV-1.2 (BoHV-1.2a and BoHV-1.2b), and BoHV-1.3 (also designated as BoHV-5) (23, 24). The subtypes BoHV-1.1 and BoHV-1.2a are typically associated with IBR and reproductive disorders, such as abortion, in infected animals. While infection with BoHV-1.2b strains usually leads to sexually transmitted disease that primarily affects the genital tract of cattle (24).

Due to the widespread used of the BoHV-1 modified-live virus (MLV) vaccines, recombinant strains derived from both field strains and MLV vaccines have been reported globally. For instance, Offay et al. isolated a novel recombinant BoHV-1 strain from an aborted fetus (25). The majority of its genome exhibited 100% sequence similarity to the field strain, while other regions shared higher homology to the MLV strain (25). Furthermore, the co-prevalence with BoHV-1 and BoHV-5 has increased the risk of genetic recombination between these viruses (26–28). For example, Maidana et al. conducted a phylogenetic analysis based on the gB and gD gene sequences of three isolates (26). The gB gene sequences of three recombinant strains were clustered into the same branch with that of those of BoHV-1 strains, whereas their gD genes showed higher sequence similarity to those of BoHV-5 strains (26).

## 3 Current diagnostic techniques for BoHV-1 detection

At present, multiple detection methodologies have been developed for the identification of BoHV-1, which can be broadly categorized into three main groups: (1) virus isolation and transmission electron microscopy (TM) techniques aimed at the direct visualization of viral particles; (2) molecular diagnostic approaches targeting viral nucleic acids, including conventional polymerase chain reaction (PCR), real-time PCR, loop-mediated isothermal amplification (LAMP), and recombinase polymerase amplification (RPA); (3) serological assays designed to detect specific antibodies against BoHV-1, such as enzyme-linked immunosorbent assay (ELISA). These representative detection methods are summarized in Table 1.

**Table 1:** Summarized diagnostic techniques for the detection of BoHV-1.

Targets	Methods	Sensitivity	Main characteristics	References
DNA genome	Conventional PCR Targeting gD gene	1 PFU/mL or 5 × 10^5^ TCID_50_	This approach enables the differentiation and diagnosis of BoHV-1, PRV, and BoHV-5.	(35)
	PCR combined with REA Targeting UL39 and US3 genes	No mentioned	This novel approach demonstrated the capacity to distinguish among BoHV-1.1, BoHV-1.2a, and BoHV-1.2b.	(36)
	GeXP-multiplex PCR assay Targeting gB gene	100 copies/μL1000 copies/μL	This method could be utilized for the detection of BTV, FMDV, IBRV, BoHV-1, VSV, and BVDV. This method demonstrated 100% agreement with the real-time PCR assay.	(39)
	Multiplex RT-PCR and automated microarray Targeting gB gene	6,800 TCID_50_/mL	This approach demonstrated the capability to detect and distinguish seven cattle viruses species including BoHV-1, which exhibited 100% agreement with conventional PCR method.	(38)
	Multiplex RT-PCR method Targeting gH gene	1,000 copies/μL	This approach was capable of identifying BoHV-1, BVDV, and AKV.	(37)
	Real-time PCR Targeting gB gene	10 copies/μL	Which exhibited high sensitivity and specificity.	(41)
	Real-time PCR Targeting gC and bCH gene	0.21 TCID_50_	This method demonstrated 100% sensitivity and specificity compared to the OIE recommended real-time PCR.	(42)
	EvaGreen-based multiplex real-time PCR assay Targeting gB, gE, and bGH genes	10 copies/μL	This technique enabled the differentiation between the BoHV-1 infected and vaccinated animals (DIAV) with a high degree of sensitivity.	(43)
	Multiplex real-time PCR method Targeting gB gene	8.26 copies/μL	This method enabled the simultaneous detection of BoHV-1, BRSV, BPIV-3, and BVDV, with the entire process being completed within 90 min.	(44)
	One-step Multiplex real-time PCR method Targeting gE gene	100 copies/μL	This method detected BoHV-1, BRSV, BVDV, BPIV-3, and IDV simultaneously while also identifying DIAV in clinical samples.	(46)
	Multiplex real-time PCR assay Targeting BHoV-1 genome	26 copies/μL	This method could simultaneously detect *Moraxella bovis*, *Moraxella bovoculi, Mycoplasma bovis*, *Mycoplasma bovoculi* and BHV-1.	(47)
	LAMP assay Targeting gC gene	10 fg/μL or 0.2 TCID_50_	This method demonstrated 100-fold greater sensitivity than conventional PCR, and the assay was completed within 90 min, with results visible to the naked eye.	(50)
	LAMP assay Targeting gE and gB genes	100 fg/μL	This method exhibited a 10-fold sensitivity improvement over conventional PCR. The approach also successfully detected DIAV in clinical samples.	(51)
	Multiple LAMP PCR assay Targeting gB gene	200 copies/μL	This approach detected and distinguished four BoHV-1 and *Mycoplasma bovis,* with results visible to the naked eye.	(52)
	RPA combined with LFD Targeting UL52 gene	5.00 copies/μL	This procedure was conducted at 38 °C for a duration of 25 min, and the amplification outcomes were directly observable on LFD strips.	(54)
	Multiple RPA assay Targeting gB gene	10,000 copies/μL	This novel approach demonstrated the capacity to distinguish BoHV-1 and BVDV, which could be conducted at 37 °C for a duration of 30 min.	(55)
	RPA combined with LFD Targeting gE gene	1 TCID_50_	This novel approach demonstrated the capacity to distinguish BoHV-1 and BoHV-5, and the amplification outcomes were directly observable on LFD strips.	(56)
	RPA combined with VF Targeting TK gene	38 copies/μL 10.9 TCID_50_	This procedure could be completed within 35 min, the detection results of which exhibited 100% agreement with fluorescence quantitative PCR.	(57)
	Polymerase spiral reaction Targeting gD gene	23 fg/μL	This procedure was conducted at 64 °C for 75 min, the results could be visible to the naked eye.	(59)
Specific antibody	Virus neutralization test Targeting neutralizing antibody	Not mentioned	This approach was regarded as the gold standard for detecting viral neutralizing antibodies, despite being complex, time-consuming, and costly.	(29)
	Indirect ELISA Targeting gB antibody	Comparable to commercial ELISA kits	Which demonstrated 100% concordance for positive samples and 93.4% concordance for negative samples compared to a commercial ELISA kit (IDEXX)	(61)
	Indirect ELISA Targeting gD antibody		Which exhibited 93.3% agreement with commercial kits.	(62)
	Latex agglutination test	Not mentioned	Which was capable of detecting antibodies against BoHV-1 and BoHV-5, and exhibited high agreement with serum neutralization test.	(39)
	Vanti™ biosensor approach	Not mentioned	This assay could be completed within 15 min, and the results were observed by the naked eye. Which also demonstrated high agreement with commercial ELISA kits.	(68)
	Solt Bot technology with nanogold labeling	Not mentioned	Which enabled direct visual interpretation of results, its sensitivity and specificity closely aligned with these of commercial ELISA kits.	(69)
	Time-resolved fluorescent immunoassay strip	1,000 TCID_50_/mL	Which could be completed within 15 min, with results discernible by the naked eye. This method demonstrated a 95.42% concordance with the fluorescence quantitative PCR assay.	(70)

### 3.1 Virus isolation and transmission electron microscopy

#### 3.1.1 Virus isolation

Virus isolation is widely considered the “gold standard” for pathogen identification and serves as a critical foundation for subsequent research endeavors, including investigations into viral infection mechanisms, antiviral drug screening, and vaccine development. A critical procedure for BoHV-1 isolation involves the collection of fresh clinical samples exhibiting a high viral load, particularly from the upper respiratory tract (e.g., nasal swab) and reproductive tract tissues of cattle infected with BoHV-1. Bovine testicular cells and Madin-Darby bovine kidney (MDBK) cells demonstrate susceptibility to BoHV-1 infection, rendering these cell lines frequently employed for the purposes of virus isolation, purification, and identification.

In brief, the supernatants derived from clinical samples were incubated with a monolayer of susceptible cells, and typical cytopathic effects will be observed within 48 to 72 h post-infection. Following this incubation period, both the cells and supernatants are collected for freeze–thaw cycles and centrifugation (29). After purification, further experiments are required to identify the isolated virus, these identification methods encompass immunofluorescence assays (IFA), molecular and serological testing, as well as transmission electron microscopy.

#### 3.1.2 Transmission electron microscopy

Transmission electron microscopy (TM) has been extensively employed to directly examine the morphological characteristics of viral particles (30, 31). In this procedure, cells infected with BoHV-1 or supernatants containing BoHV-1 are stained overnight with 2% phosphotungstic acid, and subsequently analyzed using EM. The viral particles are characterized by a roughly spherical shape, with diameters approximately between 150 and 200 nm. Moreover, the BoHV-1 particle comprises an icosahedral capsid core enveloped by a lipid membrane (30).

In addition to BoHV-1, other *herpesviruses* can infect cattle, including other subtypes of BoHV and pseudorabies virus (32, 33). These *herpesviruses* exhibit similar morphological characteristics and therefore cannot be directly identified by TM. Consequently, additional techniques such as PCR and IFA should be used in combination for accurate identification.

### 3.2 Molecular diagnostic techniques

#### 3.2.1 Conventional PCR

In the past few years, conventional PCR technique has been widely employed for the molecular identification of pathogens. In general, PCR methods usually target the conserved regions of BoHV-1 genome, such as gD, gB, gE, and gC genes. Specifically, these positive PCR products can be sequenced for further analyzing its genotype and genetic variations (23, 34). Wang et al. developed a PCR method targeting the gD gene of *herpesviruses*, enabling the simultaneous differentiation and diagnosis of the gD genes of BoHV-1 (372 bp), BoHV-5 (206 bp and 440 bp), and PRV (303 bp) (35). The sensitivity of this assay for detecting BoHV-1 nucleic acid was determined to be 1 PFU/mL or 5 × 10^5^ TCID_50_. These findings demonstrate that this PCR method possesses high specificity and sensitivity, allowing for the effective discrimination of BoHV-1, BoHV-5, and PRV in clinical samples (35).

BoHV-1 is classified into three sub-genotypes: BoHV-1.1, BoHV-1.2a, and BoHV-1.2b (36). A novel methodology has been established to enable rapid subtyping of BoHV-1 strains (36). In brief, gene fragments from UL39 (439 bp) and US3 (700 bp) are amplified by a standard multiplex PCR method. The resulting PCR products are then subjected to digestion with the *HindIII* restriction enzyme, to produce distinct fragment patterns (36). These fragment profiles allow for differentiation among the BoHV-1.1, BoHV-1.2a, and BoHV-1.2b sub-genotypes based on the number and size of the PCR fragments generated (36).

In recent years, a variety of multiplex RT-PCR based techniques have been developed to simultaneously detect BoHV-1 alongside other pathogens (37–40). Zhang et al. developed a multiplex RT-PCR method capable of identifying BoHV-1, BVDV, and Akabane virus (AKV), achieving a detection limit of 1,000 copies/μL for BoHV-1 (37). Similarly, Lung and his colleagues designed an RT-PCR assay integrated with a novel automated microarray system to concurrently detect BoHV-1 and seven additional pathogens, while the sensitivity of this approach for BoHV-1 was notably lower than conventional PCR method (38). Fan et al. successfully developed a GeXP-multiplex PCR assay for the simultaneous identification of six bovine viruses, including BoHV-1. Specifically, this innovative technology demonstrated a detection limit of 10 copies/μL for BoHV-1 (39).

#### 3.2.2 Real-time PCR

Real-time PCR constitutes an advanced detection technology evolved from traditional PCR methodologies, enabling a transition from qualitative to quantitative analysis of viral nucleic acids, thereby providing enhanced sensitivity (40). This technique is extensively used for the early detection of pathogens, contributing significantly to the prevention and control of infectious diseases (41–44). Wang and colleagues established a TaqMan real-time fluorescence quantitative PCR assay targeting the gB gene of BoHV-1, which achieved a detection sensitivity of 10 copies/μL and demonstrated no cross-reactivity with other viruses. Application of this method to 183 clinical samples resulted in the identification of six BoHV-1 positive cases (41). In a comparable study, Pawar et al. developed an EvaGreen-based multiplex real-time PCR assay for simultaneously detecting the viral genes gB and gE, along with an internal positive control gene, which was capable of differentiating between wild-type and gE-deleted strains of BoHV-1 (43).

In clinical settings, BoHV-1 often co-infects alongside other pathogens, presenting with non-specific clinical manifestations that complicate differential diagnosis. Consequently, the development of multiplex fluorescent PCR assays are capable of simultaneously detecting BoHV-1 and other pathogens, which greatly reduce diagnostic costs and enhance detection efficiency (44–48). For example, Jiang et al. established a multiplex real-time PCR assay designed for the simultaneous identification of BoHV-1, BRSV, BPIV-3, and BVDV (44). This assay demonstrated detection limits of 8.26 copies/μL for BoHV-1, BVDV, BPIV-3, and 82.68 copes/μL for BRSV. Furthermore, this method exhibited high reproducibility and specificity, thereby providing robust technical support for the rapid detection of multiple pathogens (44).

#### 3.2.3 Loop-medical isothermal amplification

Loop-medical isothermal amplification (LAMP) technology, developed by Notomi et al. enables nucleic acid amplification at constant temperatures. Unlike conventional PCR, this method eliminates the need for thermal cycling and reduces equipment complexity (49). LAMP system employs two or three pairs of primers targeting viral conserved regions, amplifying DNA fragments within nearly 1 h under isothermal conditions (60 ~ 65 °C). The amplification products generate magnesium pyrophosphate precipitates, enabling direct visual detection without specialized instrumentation (49).

Pawar et al. designed three primer pairs targeting the conserved region of the gC gene using PrimerExplorer V4 software to develop a LAMP assay for BoHV-1 nucleic acid detection (50). The assay achieved amplification within 90 min at 63 °C and detected as few as 10 viral genome copies, demonstrating 100-fold greater sensitivity than conventional PCR, while matching real-time PCR performance (50). Since most commercial attenuated and inactivated vaccines lack the gE gene, Pawar et al. also developed a LAMP assay targeting the gB and gE genes, which could be used to distinguish wild-type infections from animals vaccinated with gE-deleted vaccines (51). This assay exhibited a detection limit of 100 fg, representing a tenfold improvement over conventional PCR method, and its products could be visually assessed without requiring gel electrophoresis equipment (51).

Similar to multiplex real-time PCR, researchers designed two primers targeting the BoHV-1 gB gene and the *Mycoplasma bovis* uvrC gene. The gB gene internal primers were conjugated with FAM fluorescent probes, while the uvrC gene primers were labeled with CY5 probes (52). This assay detected the recombinant plasmid containing both target genes at 200 copies/μL, with amplification products showing distinct green fluorescence for BoHV-1 and red fluorescence for *M. bovis* (52). Clinical validation against WHO-recommended PCR methods revealed 95% ~ 96.6% sensitivity and 100% specificity, confirming its diagnostic utility (52).

#### 3.2.4 Recombinase polymerase amplification

RPA is an isothermal nucleic acid detection approach that utilizes the recombinases, single-stranded binding proteins, and DNA polymerases to amplify target sequences at constant temperatures (53). This method could be accomplished in approximately 25 min and achieve higher efficiency than loop-mediated isothermal amplification (LAMP) (53). Hou et al. established an RPA assay targeting the conserved UL52 gene region of BoHV-1, combining it with lateral flow dipsticks (LFD) to create an RPA-LFD detection system (54). After incubating nucleic acid samples in the RPA system at 38 °C for 25 min, the amplification products subsequently were directly visualized on LFD strips (54). The system detected as few as 5 copies/μL and exhibited no cross-reactivity with related *herpesviruses* or bovine pathogens. Clinical evaluations demonstrated perfect agreement with real-time PCR results, confirming its high specificity, sensitivity, and practicality (54). Jiang et al. developed a multiple RPA assay using primers targeting the gB gene of BoHV-1 and the 5’UTR gene of BVDV, enabling simultaneous detection of both viruses (55). This multiplex assay produces results within 30 min at 37 °C, with the amplification products analyzed by agarose gel electrophoresis, achieving detection limits of 10 copies/μL for BVDV and 10,000 copies/μL for BoHV-1 (55).

In addition, Wu et al. developed an RPA assay for the simultaneous detection of BoHV-1 and BoHV-5, integrating the RPA amplification products with LFD test strips to create an RPA-LFD technology (56). This approach achieved visual results within 30 min, demonstrating a detection limit of 1 TCID_50_ and sensitivity equivalent to conventional real-time PCR (56). Separately, researchers in Jilin University from China designed an RPA-VF system combining RPA with a closed vertical flow visualization device (VF) targeting the BoHV-1 TK gene (57). The complete procedure spent about 35 min, including DNA genome extraction (5 min), RPA amplification at 42 °C (25 min), and visualization (5 min). The assay detected as few as 38 copies/μL of recombinant plasmid and 10.9 TCID_50_ of infectious viruses (57). In addition, this detection method did not show cross reactivity with other pathogenic nucleic acids, and the detection results of clinical samples were completely consistent with those of fluorescence quantitative PCR (100.0%) (57).

#### 3.2.5 Polymerase spiral reaction

Polymerase spiral reaction (PSR) is an innovative isothermal nucleic acid amplification technique that utilizes DNA polymerase with chain displacement activity to efficiently amplify DNA fragments under constant temperature conditions. This characteristic makes it suitable for the rapid detection of pathogenic microorganisms (58). Malla et al. designed one pair of primers targeting the gD gene of BoHV-1 to establish a PSR method (59). This optimized reaction protocol involved an initial denaturation step at 95 °C for 5 min, followed by amplification at a constant temperature of 64 °C for 75 min (59). The positive PSR products appeared green after adding an appropriate amount of SYBR Green 1 dye, while the negative product were orange visually; in addition, the PSR amplification results could also be analyzed by agarose gel electrophoresis (59). Overall, the minimum detection limit of this method for target DNA was 23 fg/μL, which was consistent with the sensitivity of fluorescence quantitative PCR but 100 times higher than of traditional PCR; and this detection method exhibited no cross reactivity with other pathogens, therefore it had high specificity (59).

### 3.3 BoHV-1 antigen or antibody detection technologies

#### 3.3.1 Serum neutralization test

Serum neutralization test (SNT) remains the gold-standard method for detecting virus-specific neutralizing antibodies, which play a pivotal role in the assessment of vaccine efficacy and serological studies (60). As for BoHV-1, infections of susceptible cell lines, such as MDBK cells, results in distinctive cytopathic effects, including cellular rounding, vacuolization, and subsequent plaque formation (29). When viral particles are pre-incubated with serum samples containing BoHV-1 specific neutralizing antibodies, viral infection would be efficiently suppressed, preventing or attenuating the CPE development.

The procedure can be summarized as follows: BoHV-1 susceptible cells, such as MDBK cells, are seeded in 96-well culture plates, serial dilutions of the tested serum samples are subsequently prepared and combined with a predetermined titer of BoHV-1 virus before being added into the wells. The plates are incubated under controlled temperature conditions in a CO_2_ incubator, with daily monitoring for CPEs. The absence of BHoV-1 specific CPEs throughout the observation period indicates the presence of neutralizing antibodies against BoHV-1 in the serum sample, whereas their presence denotes a negative result. It is important to acknowledge that the virus neutralization test has several limitations, including its labor-intensive nature, extended assay duration, and the necessity for specialized laboratory equipment. Consequently, this method is less frequently employed in clinical practice for routine serological antibody detection.

#### 3.3.2 ELISA

ELISA exploits the specific interaction between antigens and antibodies to detect the target molecules in serum, with broad applications including the identification of antibodies and antigens. The immunogenic glycoproteins gD, gC, gB, gE, and gG of BoHV-1 are commonly employed as targets in the developments of ELISA assays (61–63). Wu et al. expressed the BoHV-1 gB protein in a prokaryotic expression system to establish an indirect ELISA, which demonstrated 100% concordance for positive samples and 93.4% concordance for negative samples when compared with a commercial antibody detection kit (IDEXX), thereby confirming its diagnostic reliability (61). Similarly, Liu et al. developed an indirect ELISA based on the gD glycoprotein, which exhibited 93.3% agreement with commercial kits, as well as high sensitivity, specificity, and reproducibility (62).

ELISAs targeting the gE glycoprotein have proven effective in differentiating wild-type BoHV-1 infections from responses induced by gE-deleted vaccines, rendering them particularly useful for seroepidemiological investigations (64, 65). In recent year, Chinese researchers developed a gE-blocking ELISA characterized by intra- and inter-assay variability below 10%, and a 97.7% concordance rate with serum neutralization tests during clinical validation (66). Similarly, Chowdhury established a blocking ELISA technique targeting the cytoplasmic tail of BoHV-1 gE, which proved effective for distinguishing between animals infected with field strains and those infected with or vaccinated against gE-deleted viruses (67).

#### 3.3.3 Other serological methods

In addition to ELISA, various serological detection techniques have been developed for identifying antibodies specific to BoHV-1. Fan et al. introduced a latex agglutination test capable of concurrently detecting antibodies against BoHV-1 and BoHV-5, When benchmarked against the serum neutralization test, this assay demonstrated a sensitivity of 94.68% and a specificity of 97.47%, suggesting its utility as a convenient diagnostic tools for BoHV-1 and BoHV-5 antibodies, albeit without the capacity to differentiate between these two strains (39). Crok et al. developed the Vanti™ biosensor method for the detection of BoHV-1 antibody, which exhibited specificity and sensitivity rates of 98.1 and 95.6%, respectively, relative to commercial ELISA kits (68). Notably, this approach offers reduced detection costs and a rapid turnaround time of 15 min, with results observable by the naked eye, obviating the need for costly instrumentation the thereby enhancing its practicality (68).

Japolla et al. devised an immunoassay that integrated Solt Blot technology with nanogold labeling for BoHV-1 antibody detection (69). This method afforded considerable convenience, enabling direct visual interpretation of result reliance on sophisticated equipment. Its sensitivity and specificity closely aligned with those of ELISA, indicating promising applicability in contexts lacking ELISA infrastructure (69). Additionally, Liu et al. developed a time-resolved fluorescent immunoassay strip for the detection of BoHV-1 antibody, capable of completing the assay within 15 min and achieving a sensitivity threshold of 1,000 TCID_50_/mL, with results discernible by the naked eye (70). This method demonstrated a 95.42% concordance with the fluorescence quantitative PCR assay endorsed by the WOAH, underscoring its suitability for monitoring BoHV-1 infections within dairy cattle populations (70).

## 4 Advances in vaccine development against BoHV-1

Vaccination represents the most effective strategy to combat infectious diseases such as BoHV-1. In recent years, much efforts have focused on developing various vaccines against BoHV-1, including inactivated, MLV, subunit, DNA, as well as vectored vaccines (as summarized in Tables 2, 3). The following section summarizes research progress pertaining to these vaccine classes.

**Table 2:** Various types of vaccines against BoHV-1 infection.

Vaccine type	Characteristic	Main advantages	References
Inactivated vaccines	gE-deleted	High safety without a risk of virulence reversion, this vaccine could provide complete protection against field BoHV-1 strains in cattle. (allow DIAV)	(73–76)
MLV vaccines	gE-deleted	Which could induce effective immune response and provide complete protection against field BoHV-1 strains in cattle. (allow DIAV)	(73, 79)
	gG/TK-deleted	Which exhibited superior immunogenicity relative to other inactivated vaccines, and provided complete protection against field BoHV-1 challenge.	(80, 81)
	gG/TK/gE-deleted	This vaccine demonstrated greater safety compared to the gG/TK-deleted vaccine and conferred partial protection against both BoHV-1 and BoHV-5 in cattle.(allow DIAV)	(82)
Subunit vaccines	gD-based	This subunit vaccine elicited a robust and balanced immune response and provided complete protection against a high-dose BoHV-1 challenge.	(84, 85)
	gD_34-380_-based	Which induced higher levels of viral neutralizing antibodies in both mice and cattle	(86)
DAN vaccines	gD-based	Immunization with this vaccine with adjuvants could elicit enhanced BoHV-1-specific humoral and cellular immune responses	(89)
	gD&CD40L-based	The vaccine with Montanide™ GEL01 adjuvant induced strong IgG1, IgG2, and neutralizing antibody responses. It also reduced clinical symptoms and viral shedding in nasal swabs after viral challenge in cattle.	(90)
	gB&gC&gD-based	This vaccine with PEG adjuvant induced higher neutralizing antibodies, total IgA, cytokines, and increased CD4 + and CD8 + T cell proportions in vaccinated mice.	(91)

**Table 3:** List of live attenuated recombinant vaccines against BoHV-1 infection.

Insert sites of viral genome	Insertion genes	Main characteristics	References
The TK gene of BoHV-1	The gC, gD, or gE&gI gene of PRV	Which conferred effective protection against both PRV and BoHV-1 in murine models	(94)
The TK gene of BoHV-1	The gC and gD of PRV	Vaccination with the candidate induced strong IgG2a responses against BoHV-1 in mice	(95)
The gE gene of BoHV-1	The VP1 of FMDV	The vaccine elicited neutralizing anti-BoHV-1 and anti-FMDV VP1 antibodies. (allow DIAV)	(97)
The TK gene of BoHV-1	The VP1 of FMDV	The recombinant vaccine elicited strong anti-FMDV antibodies and protected cattle from infection.	(98)
The gG gene of BoHV-1	The Gc and Gn of RVFV	A single vaccine dose induced neutralizing antibodies and cellular immunity against BoHV-1 and RVFV in both cattle and ovine (allow DIAV)	(100, 101)
The gE gene of BoHV-1	The G protein of RABV	This vaccine provided near-complete protection against lethal challenge of RABV in murine and bovine models	(102)
The gE gene of BoHV-1	The E2 of BVDV-2	This vaccine boosted neutralizing antibodies and protects cattle against BVDV-2.	(104)
The TK gene of BoHV-4	The gD of BoHV-1	This novel vaccine boosted high neutralizing antibodies against BoHV-1 in mice	(105)
The ORFs of BPSV	The gD and gB of BoHV-1	This vaccine induced BoHV-1 neutralization antibodies and provided partial protection in cattle	(106)
The E3 gene of BAdV-3	The gD of BoHV-1	Which induced high gD-specific mucosal and systemic immune responses in cattle.	(107)
The gp64 gene of baculovirus	The gD of BoHV-1	Which elicited elevated levels of neutralizing antibodies against BoHV-1 in murine models.	(108)

### 4.1 Inactivated and modified-live virus vaccines

*Alphaherpesvirinae* gE protein is critically involved in viral infection progresses, immune evasion, and host cell interaction (71, 72), thus which has become a significant target antigen in the development of gene-modified vaccines, including inactivated and MLV vaccines. Notably, inactivated vaccines demonstrate excellent safety without potential risks of reversion to virulence, and which could provided efficient protein against BoHV-1 in water buffalo and cattle (73–76). Thus inactivated vaccines are widely used for the eradication of BoHV-1 in some Europe countries (65).

Currently, several MLV vaccines are commercially available and have substantially contribute to mitigating the economic impact associated with BoHV-1 infection (77, 78). Moreover, numerous MLV vaccine candidates have been enveloped, demonstrating effective protection against BoHV-1 infection (73, 79–82). For instance, Weiss et al. engineered a gE-deleted BoHV-1 strain expressing green fluorescent protein, and intramuscular administration of this genetically modified strain significantly enhanced the production of virus-neutralizing antibodies (80). Similarly, Zhang et al. developed a BoHV-1 strain with deletions in the gG and TK genes, immunization with this novel vaccine candidate elicited high titers of specific gB antibodies and neutralizing antibodies (80, 81). Furthermore, vaccination with this gG-/TK- gene-deleted vaccine strain conferred robust protection against high challenge with field BoHV-1 strain, and exhibited superior immunogenicity relative to other inactivated vaccines (80, 81). Subsequently, they constructed a recombinant BoHV-1 gG-/TK−/gE- triple deleted strain, which exhibited lower replication efficiency compared to the wild-type strain (82). Moreover, this vaccine candidate was safer to calves compared to BoHV-1 gG&TK gene-deleted strain, while which exhibited lower protection efficiency against wild-type BoHV-1 and BoHV-5 strains (82).

### 4.2 Subunit vaccines

Subunit vaccine constitutes a form of immunization developed through the isolation of distinct immunogenic antigens, including proteins, polysaccharides, or lipids, derived from pathogenic organisms (83). In contrast to the inactivated vaccines or MLV vaccines, subunit vaccines exclude the complete pathogen and instead employ specific structural components to elicit a target immune response and induce protective immunity (84).

BoHV-1 possesses a large DNA genome that encodes several envelope proteins. Among these, gD, gC, and gB have been identified as principle candidates for the development of subunit vaccines. Previous studies demonstrated that immunization with a truncated, secreted form of BoHV-1 gD combined with CpG oligodeoxynucleotides elicited a robust and balanced immune response in calves (84), which provided complete protection against a high-dose BoHV-1 challenge (85). Recently, Hoa et al. employed both baculovirus and *Escherichia coli* systems to produce the ectodomain of gD (gD_34-380_), designated as EgD and BgD, respectively (86). Immunization with BgD induced higher levels of viral neutralizing antibodies in mice compared to both the EgD and inactivated vaccine groups. Furthermore, the BgD subunit vaccine was able of eliciting robust neutralizing antibody responses against BoHV-1 in cattle (86). Similarly, recombinant BoHV-5 gD expressed in the *Pichia pastoris* system could generate elevated neutralizing antibody titers against both BoHV-5 and BoHV-1 (87).

### 4.3 DNA vaccines

DNA vaccines are developed by inserting the gene encoding a specific antigen into a plasmid vector, thereby enabling the expression of target antigen within eukaryotic cells (88). This vaccination strategy offers multiple advantages, such as straightforward design and production, cost efficiency, and favorable biosafety profiles (88). Langellotti et al. employed the plasmid pRSET-B to generate a recombinant plasmid encoding a truncated, secreted form of gD (PCIgD) (89). Immunization with pCIgD, combined with various adjuvants elicited enhanced BoHV-1-specific humoral and cellular immune responses compared to the pCIgD only group in murine models (89). Next, they constructed a recombinant plasmid that encoded both gD and the functional extracellular domain of bovine CD40 ligand (pCIgD-CD40L) using the pCIgD plasmid (89). Administration of pCIgD-CD40L alongside the adjuvant Montanide™ GEL01 resulted in increased levels of total IgG1, IgG2, and neutralizing antibodies against BoHV-1 in bovine serum (90). Furthermore, this immunization regimen significantly alleviated clinical symptoms and reduced viral shedding in nasal swabs following viral challenge in cattle (90).

In addition to gD, Liu et al. combined plasmids encoding gB, gC, and gD proteins, adsorbed onto PEI magnetic beads, and demonstrated that vaccination with this mixture and the adjuvant polyethylene glycol have induced the expression of viral proteins in the lung but not the spleen of mice (91). This approach also elicited elevated levels of neutralizing antibodies, total IgA, cellular cytokines and the increased proportions of CD4 + and CD8 + T cells in vaccinated mice (91).

### 4.4 Vectored vaccines developed using BoHV-1

Consistent with other members of the *Alphaherpesvirinae* subfamily, BoHV-1 possesses a large DNA genome that includes several non-essential genes, such as gE, TK, and gG. The deletion of these genes does not compromise viral replication efficiency (92). Consequently, the successful incorporation of exogenous genes into these non-essential genomic regions of BoHV-1, coupled with the efficient selection of recombinant viruses via homologous recombination techniques, indicating that BoHV-1 holds potential as a novel vaccine vector (92).

#### 4.4.1 Recombinant vaccine candidate for management of BoHV-1 and PRV

Pseudorabies virus (PRV) is considered a significant infectious agent posing a substantial threat to the development of the global swine industry (93). Furthermore, its ability to transmit across species, from pigs to other animals such as cattle and goats, has garnered considerable scientific interest (93). Otsuka et al. developed recombinant BoHV-1 strains that expressed the gB, gC, gD, or gE of PRV (94). Among these, recombinant strains expressing gC, gD, gE and gI but not gB, conferred effective protection against both PRV and BoHV-1 in murine models (94). Takashima et al. successfully incorporated the PRV gB and gC genes into the BoHV-1 genome, this recombinant strain demonstrated increased susceptibility to mouse tissue cell lines compared to the parental BoHV-1 strain (95). Moreover, immunization with this vaccine candidate induced robust IgG2a antibody responses against BoHV-1 in mice (95).

#### 4.4.2 Recombinant vaccine candidate for management of BoHV-1 and FMDV

Foot-and-mouth disease virus (FMDV) represents a significant challenge to the global livestock industry, and its widespread prevalence also poses a potential threat to human populations (96). Ren et al. constructed a recombinant BoHV-1 strain engineered to express the VP1 protein of FMDV (97). Further experiments conducted in rabbits demonstrated that this vaccine candidate elicited robust viral neutralizing antibody response against BoHV-1, as well as generated specific antibodies targeting the FMDV VP1 protein (97). Kit et al. developed a recombinant BoHV-1 vector expressing the VP1 protein of FMDV, as verified by western blot analysis (98). Immunization with this recombinant vaccine induced high levels of IgG and neutralizing antibody responses against FMDV, conferring effective protection against FMDV infection in cattle (98).

#### 4.4.3 Recombinant vaccine candidate for management of BoHV-1 and RVFV

Rift valley fever virus (RVFV) infection is associated with elevated mortality rates neonatal animals and induces abortion in pregnant livestock, such as cattle and sheep (99). Moreover, the prevalence of RVFV poses potential threats to human health (99). Pavulraj et al. developed a BoHV-1qmv-vectored vaccine expressing the amino-terminal glycoprotein (Gn) and carboxy-terminal glycoprotein (Gc) of RVFV (100). Notably, this recombinant strain demonstrated significant attenuation and safety in cattle (100, 101). A single vaccination dose of the vaccine elicited strong, and specific neutralizing antibody responses against both BoHV-1 and RVFV, in addition to inducing a cellular immune response (100). Similarly, this vaccine candidate has the potential to generate a strong humoral and cellular immune response against both BoHV-1 and RVFV in ovine subjects (101).

#### 4.4.4 Recombinant vaccine candidate for management of BoHV-1 and RV

Rabies virus (RABV) has been endemic in over 150 countries globally, posing a significant risk to humans as well as various animal species, including livestock such as cattle and sheep (102). Zhao et al. constructed a recombinant BoHV-1 strain that expressed the RABV glycoprotein (G) while simultaneously deleting the gE (102). Importantly, the RABV G was located on the surface of this recombinant viral particles. A single administration of this novel vaccine candidate elicited robust activation of dendritic cells and B cells, and a specific neutralizing antibody response against RABV (102). This immune response conferred near-complete protection against a severe lethal challenge infection in both murine and bovine models (102).

#### 4.4.5 Recombinant vaccine candidate for management of BoHV-1 and BVDV

Bovine viral diarrhea virus (BVDV) and BoHV-1 are significant pathogens contributing to the bovine respiratory disease complex on a global scale (103). Zhao et al. generated a recombinant BoHV-1 strain with quadruple gene mutations that concurrently expressed the E2 protein of BVDV-2 (104). Further analysis demonstrated that this novel vaccine candidate have elicited significantly elevated levels of neutralizing antibodies targeting BoHV-1 and BVDV-2, in comparison to the commercially available vaccines (104). Also, which could confer protection to the cattle against a virulent BVDV-2 challenge (104).

#### 4.4.6 Vectored vaccines developed using other DNA viruses

In addition to BoHV-1, several large DNA viruses have been utilized as vectors to express the gD and/or gB proteins of BoHV-1, including BoHV-4, Bovine popular stomatitis virus (BPSV), bovine adenovirus-3 (BADV-3), and baculovirus (105–108). Notably, Delhon et al. constructed two recombinant BPSV strains expressing either the gD protein alone or both the gD and gB proteins of BoHV-1, respectively (107). Both of two vaccine candidates have induced robust neutralizing antibody responses against BoHV-1 in cattle, and conferred approximately 75% protection following challenge with high doses of BoHV-1 (107).

## 5 Perspective and conclusion remarks

BoHV-1 continues to be a primary infectious agent responsible for IBR, reproductive disorders and immunosuppression in cattle, resulting in significant economic losses worldwide. A meta-analysis conducted by Chen et al. evaluated the prevalence of BoHV-1 in China over the period from 1979 to 2018, revealing a pooled prevalence rate of 40.37% (17,535/43441) within cattle populations (109). González et al. reported a seroprevalence of BoHV-1 infection of 44.9% (325/725) in Colombia (11). Moreover, 21.43% (1,130/5273) of unvaccinated animals tested positive for BoHV-1 specific antibodies in samples collected from Ireland between 2018 and 2020 (110). Collectively, these findings indicate a substantial prevalence of BoHV-1 infection within cattle populations globally. In response to this challenge, numerous diagnostic techniques and vaccines have been developed, which are crucial for the effective management for this disease and the mitigation of its adverse impacts.

In recent years, a range of diagnostic methodologies has been established for the detection of specific antibodies, nucleic acids, or antigens of BoHV-1. These advancements have significantly enhanced researcher’s ability to elucidate the global epidemiological patterns of BoHV-1. However, this section presents several concerns. Firstly, despite the development of numerous detection methods, most were designed to target gene or antibodies other than the gE gene of BoHV-1. It is recommended that detection techniques specifically targeting the gE nucleic acid or antibodies be developed to enable differentiation between wild-type infections and animals vaccinated with gE-deleted vaccines. Secondly, considering the infrastructure constraints in many cattle farming regions, these practical, reliable, and cost-efficient DNA assays (such as specific LAMP or RPA) can be effectively deployed at the farm level by non-specialist personnel to facilitate regional eradication initiatives (53, 58).

In terms of the progress in vaccine development, the primary categories encompass inactivated vaccines, MLV vaccines, subunit vaccines, DNA vaccines, and live virus-vectored vaccines. Importantly, these vaccine types have demonstrated effective protection against BoHV-1 in animal models. However, several concerns are summarized in this section Firstly, virus-vectored vaccines have demonstrated significant potential in the prevention of various infectious diseases, including BoHV-1 infection. However, the strong immune response elicited by the host against the BoHV-1 vector itself may constrain the efficacy or durability of the immune response directed toward the foreign antigen following subsequent administrations (111). Secondly, similar with other animal herpesvirus, attenuated MLV vaccines incorporating deletions of one or multiple genes are considered safe and have the potential to confer effective immunity against BoHV-1 infection. However, the potential risk of genetic reversion to virulence or recombination with wild-type field strains remains a concern that cannot be overlooked (112). Thirdly, mucosal immunity is integral to the antiviral defense against BoHV-1 infection, particularly because BoHV-1 predominantly causes respiratory disease. Nevertheless, the capacity of these novel vaccines to elicit mucosal IgA antibody responses in cattle remains uninvestigated. Or the existing relationship between neutralizing antibody titers and effective mucosal protection has yet to be thoroughly examined.

In conclusion, BoHV-1 remains a significant pathogen affecting cattle globally, with high prevalence rates reported across different regions. Although various diagnostic tools and vaccines have been developed to combat the disease, challenges remain in differentiating infected from vaccinated animals and in ensuring accessibility in resource-limited settings. Targeting the gE gene in diagnostic assays could enhance this differentiation, while portable, rapid detection technologies such as RPA-LFD or biosensors offer practical solutions for field use. Vaccine development has progressed with promising candidates like live virus-vectored vaccines showing potential for multi-disease protection. However, issues related to cost, safety, and efficacy hinder their commercialization. Future research should focus on optimizing vaccine formulations, reducing production costs, and expanding vaccine applications to other susceptible species to better control BoHV-1 transmission and its economic impact.
